# Environmental Isolate of *Rahnellaaquatilis* Harbors Class 1 Integron

**DOI:** 10.1007/s00284-015-0917-4

**Published:** 2015-09-30

**Authors:** Ryszard Koczura, Joanna Mokracka, Nicoletta Makowska

**Affiliations:** Department of Microbiology, Faculty of Biology, Adam Mickiewicz University in Poznań, ul. Umultowska 89, 61-614 Poznań, Poland

## Abstract

The paper presents first description of class 1 integron in an environmental strain of *Rahnella aquatilis*, a rarely isolated Gram-negative bacterium of the family *Enterobacteriaceae.* The strain was isolated from the Warta river water, Poland. Class 1 integrase gene was detected by a PCR assay. Sequencing of the integron’s variable region showed the presence of a *dfrA1-aadA1* gene cassette array. The integron was located in a 54-kbp plasmid that was transferable to *Escherichia coli* J-53 recipient strain in a conjugation assay. The integron-bearing *R*. *aquatilis* strain was resistant to aminoglycosides, penicillins, trimethoprim, sulfamethoxazole, and trimethoprim/sulfamethoxazole. This paper confirms that water environment play a major role in the spread of integrons and, consequently, antimicrobial resistance, among bacteria of various genera.

## Introduction

*Rahnella aquatilis* is a rarely isolated Gram-negative bacterium of the family Enterobacteriaceae. *Rahnella aquatilis* occurs mainly in freshwater; however, it is occasionally found in foods and human clinical specimens [6]. Clinical isolates have been cultured mostly from immunocompromised hosts [3]; however, a case of sepsis in a healthy patient has been described as well [3].

Due to limited number of clinical cases and studies on antimicrobial susceptibility of environmental isolates, there is little data about antimicrobial resistance patterns and mechanisms of *R*. *aquatilis*. Resistance to amoxicillin, ampicillin, ticarcillin, cefotaxime, and cephalothin has been reported [3, 14, 17]. *Rahnella aquatilis* can also produce a class A extended-spectrum β-lactamase [14].

Mobile integrons play an important role in the spread of antibiotic resistance. They are responsible for integration and rearrangements of resistance determinants called gene cassettes. An integron consists of an integrase gene, a primary recombination site called *attI*, and a promoter P_C_ that directs transcription of the integrated genes. Classes of integrons are distinguished upon the sequence of integrase gene. The most frequent are class 1 integrons that are considered to play the main role in the emergence and widespread of resistance genes [2].

Integrons have been detected in numerous Gram-negative species, including those of the family Enterobacteriaceae [12]. The aim of this study was to characterize a class 1 integron and corresponding antimicrobial resistance in an environmental *R*. *aquatilis* strain isolated from river water.

## Materials and Methods

### Bacterial Strain

A *R*. *aquatilis* strain designated MPU 658/3 was used in the study. It was cultured from the water of the Warta river taken in Sieradz, Poland. The strain was isolated on Brilliance E. coli/Coliform Selective Medium (Oxoid) and identified using multilocus sequence analysis (MLSA) of *atpD*, *gyrB*, *infB*, and *rpoB* according to Brady et al. [1]. *Rahnella aquatilis* CIP 78-65^T^ type strain was used as reference.

### Detection of Integrase Genes

The presence of integron integrases in was determined using multiplex PCR method according to Dillon et al. [4] with primers complementary to class 1, 2, and 3 integrase genes. The amplification involved an initial denaturation (94 °C, 5 min), followed by 30 cycles of denaturation (94 °C, 1 min), annealing (59 °C, 1 min), and extension (72 °C, 1 min), with a final extension step at 72 °C for 8 min. *Escherichia coli* AMU 2036/6 [8] and D1/7 [13] strain were used as positive controls for class 1 and class 2 integrase genes, respectively. *Escherichia coli* K12 was used as negative control.

### Analysis of the Variable Region of Class 1 Integron

Variable part of the class 1 was amplified using primers complementary to its 5′ and 3′ conserved regions [9]. PCR amplification was conducted as follow: initial denaturation at 94 °C for 5 min, and 30 cycles of 94 °C for 1 min., 55 °C for 1 min., 72 °C for 5 min., and final elongation at 72 °C for 8 min. The amplicon was sequenced in a 3130xl Genetic Analyzer (Applied Biosystems). Sequence data were assembled with DNA Baser (HeracleSoftware) and aligned with available GenBank data using Nucleotide basic local alignment search tool (BLAST). A gene cassette was identified if the similarity with GenBank data was equal or higher than 95 %.

All PCR reactions were done in a C1000 Touch thermal cycler (Bio-Rad). The products were separated in 1.5 % agarose gel. Molecular weight of PCR products was determined with GelCompar II 3.5 software (Applied Maths). All experiments were done in duplicate.

### Antimicrobial Susceptibility Testing

Antimicrobial susceptibility was determined with the standard disk diffusion method according to The European Committee on Antimicrobial Susceptibility Testing guidelines, with the 4.0 version of breakpoint tables for interpretation of zone diameters [5]. The following antimicrobials were used: amikacin (30 μg), gentamicin (10 μg), netilmicin (10 μg), tobramycin (10 μg), ampicillin (10 μg), piperacillin (30 μg), piperacillin/tazobactam (36 μg), amoxicillin/clavulanic acid (30 μg), cefepime (30 μg), cefotaxime (5 μg), ceftazidime (10 μg), imipenem (10 μg), aztreonam (30 μg), ciprofloxacin (5 μg), norfloxacin (10 μg), chloramphenicol (30 μg), streptomycin (10 μg), trimethoprim (5 μg), and trimethoprim/sulfamethoxazole (25 μg). Production of extended-spectrum β-lactamases (ESBL) was checked by double-disk synergy test with ceftazidime, cefotaxime, and amoxicillin/clavulanic acid. All antibiotic disks were provided by Oxoid. *Escherichia coli* ATCC 25922 was used as a control strain.

The presence of *bla*_TEM_ β-lactamase gene was determined by PCR assay with primers TEM-F and TEM-R according to Sáenz et al. [15]. PCR amplification was conducted as follow: initial denaturation at 94 °C for 5 min, and 30 cycles of 94 °C for 1 min., 60 °C for 1.5 min., 72 °C for 5 min., and final elongation at 72 °C for 8 min.

### Plasmid Analysis

Plasmid DNA was isolated with Plasmid Mini AX (A&A Biotechnology) kit and separated in 0.7 % agarose gel with *E. coli* V-517 plasmids used as molecular size markers. The DNA was transferred to Immobilon-NY+ membrane (Millipore) and fixed by UV cross-linking. Class 1 integrase gene was detected by digoxigenin-labeled *intI1* probes with the use of DIG DNA Labeling and Detection Kit (Roche) according to manufacturer’s instruction.

### Conjugation Assay

Integron transfer ability was determined in broth-mating conjugation assay with *E. coli* J-53 (Rif^R^) as recipient strain. Briefly, overnight cultures of the donor and recipient strain were mixed in equal volumes, grown to mid-exponential phase, plated onto Mueller–Hinton agar plates containing rifampicin and sulfamethoxazole (all donor strains were sulfamethoxazole-resistant), and incubated for 24 h at 30 °C. Transconjugants were identified phenotypically and the presence of integrons was checked by the PCR assay described above.

## Results and Discussion

Although *R*. *aquatilis* is a bacterium that naturally inhabitants freshwater ecosystems, it is capable of infecting humans, especially those immunocompromised or with an underlying chronic disease. Therefore, it is important to expand knowledge on its antimicrobial resistance and mechanisms that can lead to the spread of resistance.

The *R*. *aquatilis* MPU 658/3 strain used in our study was isolated from the water of the Warta river taken in Sieradz, Poland. PCR-screening for integrase genes showed the presence of *intI1* gene. On the contrary, the genome of *R*. *aquatilis* CIP 78-65^T^ type strain does not contain an integrase gene [11]. Class 1 integrons are present in the genomes of clinical Gram-negative strains, but are also found in environmental isolates, albeit less frequently. They have been detected in strains of numerous species of the Enterobacteriaceae family, including *Escherichia* spp., *Citrobacter* spp., *Enterobacter* spp., *Klebsiella* spp., *Kluyvera* spp., *Morganella* spp., *Pantoea* spp., *Proteus* spp., *Salmonella* spp., and *Serratia* spp. [10, 12].

**Fig. 1:**
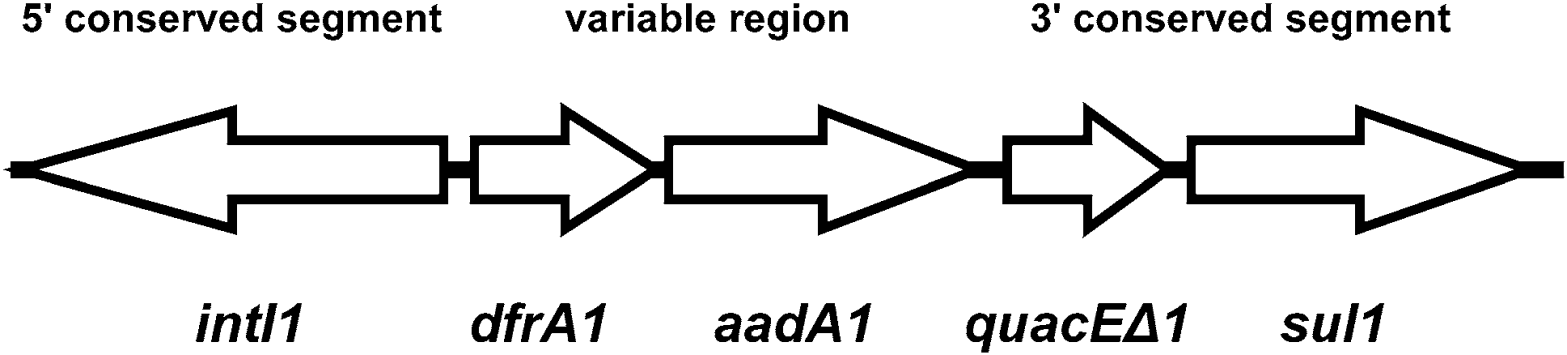
Genetic structure of class 1 integron in *Rahnella aquatilis* strain MPU 658/3

Amplification of the variable region of the class 1 integron of the *R*. *aquatilis* yielded a 1.6-kbp-long PCR product. Sequencing of the amplicon showed that it contained two genes: *dfrA1* and *aadA1* (Fig. 1). The former codes for dihydrofolate reductase conferring resistance to trimethoprim, whereas the latter encodes aminoglycoside adenylyltransferase responsible for resistance to streptomycin and spectinomycin. The *dfrA1*-*aadA1* gene cassette array is common and may be present in up to 20 % of class 1 integron-harboring isolates [12].

The *R*. *aquatilis* MPU 658/3 strain was resistant to aminoglycosides (streptomycin, and amikacin), penicillins (ampicillin, piperacillin), dihydrofolate reductase inhibitors (trimethoprim), sulfonamides (sulfamethoxazole), and trimethoprim/sulfamethoxazole. Apart from intrinsic resistance characteristic for the members of the Enterobacteriaceae family, *R*. *aquatilis* is also naturally resistant to ticarcillin and cefuroxime [16]. Although the resistance pattern was relatively narrow, it indicated multidrug-resistant phenotype typical for bacteria carrying integrons [12]. The resistance of *R*. *aquatilis* MPU 658/3 to aminoglycosides, trimethoprim, sulfamethoxazole, and trimethoprim/sulfamethoxazole was related to the class 1 integron due to the presence of *dfrA1*-*aadA* gene cassette array, and *sul1* gene in the 3′ conserved segment. Resistance to penicillins was determined by the presence of a of *bla*_TEM_ gene.

The *R*. *aquatilis* MPU 658/3 strain did not produce an extended-spectrum β-lactamase. ESBL phenotype in *R*. *aquatilis* can be mediated by RAHN-1 or RAHN-2 class A β-lactamases encoded by genes located in the chromosome [14].

The class 1 integron of *R*. *aquatilis* MPU 658/3 was located in a ~54-kbp plasmid that was transferable to *E. coli* J-53 recipient strain. The transconjugant strain was positive for the presence of class 1 integrase and was resistant to streptomycin, ampicillin, piperacillin, trimethoprim, sulfamethoxazole, and trimethoprim/sulfamethoxazole. The transferred resistance to streptomycin, trimethoprim, sulfamethoxazole, and trimethoprim/sulfamethoxazole was related to the class 1 integron due to the presence of *dfrA1*-*aadA* gene cassette array, and *sul1* gene in the 3′ conserved segment.

To sum up, we describe for the first time a class 1 integron in an environmental strain of *R*. *aquatilis*. This finding confirms that water environment play a major role in the spread of integrons and, consequently, antimicrobial resistance, among bacteria of various genera.
